# Optic chiasm measurements may be useful markers of anterior optic pathway degeneration in neuromyelitis optica spectrum disorders

**DOI:** 10.1007/s00330-020-06859-w

**Published:** 2020-04-26

**Authors:** Valentin Juenger, Graham Cooper, Claudia Chien, Meera Chikermane, Frederike Cosima Oertel, Hanna Zimmermann, Klemens Ruprecht, Sven Jarius, Nadja Siebert, Joseph Kuchling, Athina Papadopoulou, Susanna Asseyer, Judith Bellmann-Strobl, Friedemann Paul, Alexander U. Brandt, Michael Scheel

**Affiliations:** 1 Experimental and Clinical Research Center, Max Delbrueck Center for Molecular Medicine and Charité – Universitätsmedizin Berlin, corporate member of Freie Universität Berlin, Humboldt-Universität zu Berlin and Berlin Institute of Health, Berlin, Germany; 2 NeuroCure Clinical Research Center, Charité – Universitätsmedizin Berlin, corporate member of Freie Universität Berlin, Humboldt-Universität zu Berlin and Berlin Institute of Health, Charitéplatz 1, 10117 Berlin, Germany; 3 Department of Neuroradiology, Charité – Universitätsmedizin Berlin, corporate member of Freie Universität Berlin, Humboldt-Universität zu Berlin and Berlin Institute of Health, Berlin, Germany; 4 Einstein Center for Neurosciences, Berlin, Germany; 5 Department of Experimental Neurology and Center for Stroke Research Berlin, Charité – Universitätsmedizin Berlin, Berlin, Germany; 6 Multiple Sclerosis Center, Dept. of Neurology, University of California San Francisco, San Francisco, CA USA; 7 Department of Neurology, Charité – Universitätsmedizin Berlin, corporate member of Freie Universität Berlin, Humboldt-Universität zu Berlin and Berlin Institute of Health, Berlin, Germany; 8 Molecular Neuroimmunology Group, Department of Neurology, University of Heidelberg, Heidelberg, Germany; 9 Neurologic Clinic and Policlinic, Departments of Medicine, Clinical Research and Biomedicine University Hospital Basel, Basel, Switzerland; 10 Department of Neurology, University of California, Irvine, CA USA

**Keywords:** Optic neuritis, Optic chiasm, Magnetic resonance imaging, Neuromyelitis optica

## Abstract

**Objectives:**

We aimed to evaluate optic chiasm (OC) measures as potential imaging marker for anterior optic pathway damage assessment in the context of neuromyelitis optica spectrum disorders (NMOSD).

**Materials and method:**

This cross-sectional study included 39 patients exclusively with aquaporin 4-IgG seropositive NMOSD of which 25 patients had a history of optic neuritis (NMOSD-ON) and 37 age- and sex-matched healthy controls (HC). OC heights, width, and area were measured using standard 3D T1-weighted MRI. Sensitivity of these measures to detect neurodegeneration in the anterior optic pathway was assessed in receiver operating characteristics analyses. Correlation coefficients were used to assess associations with structural measures of the anterior optic pathway (optic nerve dimensions, retinal ganglion cell loss) and clinical measures (visual function and disease duration).

**Results:**

OC heights and area were significantly smaller in NMOSD-ON compared to HC (NMOSD-ON vs. HC *p* < 0.0001). An OC area *smaller than* 22.5 mm^2^ yielded a sensitivity of 0.92 and a specificity of 0.92 in separating chiasms of NMOSD-ON from HC. OC area correlated well with structural and clinical measures in NMOSD-ON: optic nerve diameter (*r =* 0.4, *p* = 0.047), peripapillary retinal nerve fiber layer thickness (*r* = 0.59, *p = 0*.003), global visual acuity (*r* = − 0.57, *p =* 0.013), and diseases duration (*r* = − 0.5, *p* = 0.012).

**Conclusion:**

Our results suggest that OC measures are promising and easily accessible imaging markers for the assessment of anterior optic pathway damage.

**Key Points:**

• *Optic chiasm dimensions were smaller in neuromyelitis optica spectrum disorder patients compared to healthy controls.*

*• Optic chiasm dimensions are associated with retinal measures and visual dysfunction.*

*• The optic chiasm might be used as an easily accessible imaging marker of neurodegeneration in the anterior optic pathway with potential functional relevance.*

## Introduction

Neuromyelitis optic spectrum disorders (NMOSDs) are inflammatory autoimmune CNS diseases that preferentially target the optic nerves and are frequently associated with serum autoantibodies to aquaporin-4 [1, 2]. Optic pathway degeneration following optic neuritis (ON) [3, 4] results in atrophy involving the entire visual pathway [5–10]. At the optic chiasma (OC), fibers from the left and the right optic nerve merge and form the site of highest axonal density. Direct damage or other pathophysiological effects affecting the optic nerves may accumulate in the OC, making it a promising target for the assessment of anterior optic pathway damage.

Measures of optic pathway degeneration are an important outcome measure of ON, since they are related to impaired visual function and reduction of vision-related quality of life [11–14]. Optic pathway dimensions as assessed by MRI have been used as a surrogate marker of inflammatory damage and atrophy of the optic nerve and anatomically connected tracts [3, 4, 15–18]. It has been suggested that MRI is sensitive to axonal loss as a cause of optic nerve atrophy [17–20]. Optical coherence tomography (OCT) reveals damage to the retinal axons and ganglion cells by means of measuring peripapillary retinal nerve fiber layer (pRNFL) thickness and combined ganglion cell-inner plexiform layer (GCIPL) volume. OCT measures have been successfully used as surrogate markers of optic nerve atrophy [16, 17, 19, 21–25], being associated to MRI-detected macro- and microstructural optic pathway atrophy and visual function [16, 17, 20, 26–28].

Although MRI is used as part of the routine clinical workup of NMOSD patients [29, 30], no method to evaluate optic pathway damage has been established. In addition to the accumulation of damage within the anterior optic pathway, the OC appears particularly promising as a potential imaging marker, since it would simplify evaluation by reducing the region of interest from multiple structures to one. Only few studies have focused on the assessment of physiologic OC dimensions [31–33] and their changes in optic atrophy [18], while quantitative correlation analysis to visual function and optic pathway degeneration has not been performed.

The aim of this study was to assess whether neurodegenerative changes in the anterior optic pathway are detectable by assessing OC measures. We hypothesized that the OC assessment in standard 3D-T1w images is sensitive to anterior optic pathway damage. To test this hypothesis, we used NMOSD as a model for optic pathway damage and compared different OC measures (area, width, left, central, right, and total height) between aquaporin 4-IgG (AQP4-IgG) seropositive NMOSD patients with and without history of ON (NMOSD-ON and NMOSD-NON) and healthy controls (HC). In addition, we investigated the association of OC measures with optic nerve diameter, visual acuity, pRNFL thickness, and GCIPL volume in NMOSD-ON.

## Material and methods

### Study population

Data of 78 NMOSD patients acquired from an ongoing longitudinal prospective observational cohort study at the NeuroCure Clinical Research Center, Charité-Universitätsmedizin Berlin (recruited from May 2013 to January 2018) were screened for eligibility. All patients (i) were 18 years or older and (ii) had a diagnosis of AQP4-IgG seropositive NMOSD according to the current panel criteria [29] and (iii) either had a last ON attack at least 5 months prior to MRI or had no history of ON. AQP4-IgG status was determined by a cell-based assay (Euroimmun, Lübeck, Germany). Patients with AQP4-IgG seronegative (*n* = 25) antibody status, unknown antibody status and/or incomplete clinical data (*n* = 10), lacking MRI data (*n* = 3), or ON within 5 months prior to MRI (*n* = 1) were excluded.

Thirty-nine patients exclusively with AQP4-IgG seropositive NMOSD and 37 age- and sex-matched HC subjects were included in this study (Table 1). All HC subjects were 18 years or older and had ophthalmologic testing and no history of neurological or ophthalmological diseases.

**Table 1:** Demographics and clinical characteristics

	HC	NMOSD-NON	NMOSD-ON	*p*
Number	37	14	25	–
Age, years (mean, SD)	47.8 (12.6)	53.8 (12.5)	48.09 (14.9)	0.33
Sex (F/M) (% female)	30/7 (81%)	14/0 (100%)	21/4 (84%)	0.22
Disease duration, years (median, range)	–	–	7.1 (3–34.1)	
Number of ON (median, range)	–	–	2 (1–12)	–
Time since first ON, years (median, range)	–	–	6.9 (3.4–32.2)	–
Time since last ON, years (median, range)	–	–	4.9 (0.5–13.2)	–
ON involvement, bilateral/unilateral	–	–	11/14 (8 right/6 left)	–
Optic nerve diameter, mm (mean, SD)	8.37 (0.50)	8.13 (0.90)	7.06 (1.23)	< 0.001
pRNFL, μm (mean, SD)	91.99 (15.10)	97.96 (10.13)	67.57 (17.92)	< 0.001
GCIPL mm^3^ (mean, SD)	1.83 (0.28)	1.88 (0.14)	1.49 (0.25)	< 0.001
Visual acuity, logMAR (mean, SD)	− 0.01 (0.21)	−0.15 (0.21)	0.30 (0.63)	0.04
Visual Functional System Score (median, range)	–	0 (0–2)	1 (0–6)	< 0.001
EDSS (median, range)	–	3 (1–7)	4 (0–6.5)	0.71

*p* values are groupwise comparisons. *HC* = healthy controls; *NMOSD-ON* = neuromyelitis optica patients with history of optic neuritis; *NMOSD-NON* = neuromyelitis optica patients without history of optic neuritis; *ON* = optic neuritis; *pRNFL* = peripapillary retinal nerve fiber layer; *GCIPL* = combined ganglion cell-inner plexiform layer; *logMAR* = logarithm of the minimum angle of resolution; *EDSS* = Expanded Disability Status Scale

This study was approved by the local ethics committee (Ethikkommission der Charité–Universitätsmedizin Berlin; EA1/131/09) and conducted according the declaration of Helsinki and applicable German law. All participants gave written informed consent.

### MRI acquisition

MRI data were acquired on the same 3-T scanner (MAGNETOM Trio, A Tim System, Siemens) at the Berlin Center for Advanced Neuroimaging using a volumetric high-resolution T1-weighted MPRAGE sequence (RT = 1900 ms, TE = 3.03 ms, TI = 900 ms, FOV = 256 × 256 mm^2^, matrix 256 × 256, slice thickness 1 mm). OC measurements were performed on reconstructed 3D MPRAGE MR images.

### Optic chiasm measures

**Fig. 1:**
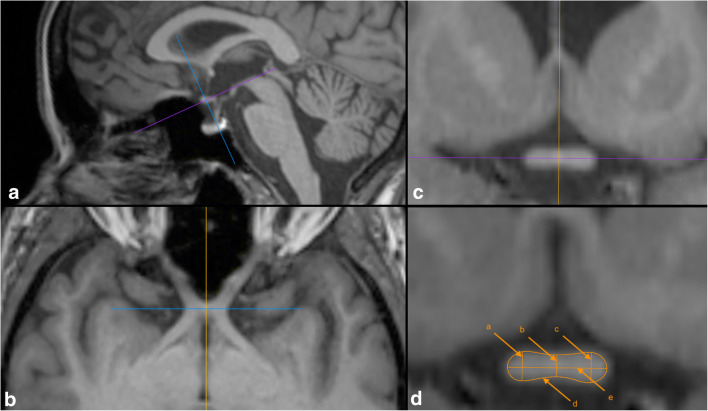
Optic chiasm measurement. Panels **a**, **b**, and **c** illustrate how axes were adjusted perpendicularly to the orientation of the OC in the center point. Panel **d** shows a sample OC measurement (a = right, b = central, c = left height, d = area, e = width)

**Fig. 2:**
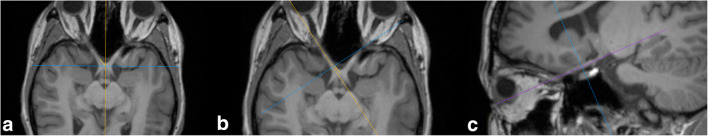
Optic nerve measurement. Panels **a**, **b**, and **c** illustrate how axes were adjusted perpendicularly to the orientation of the optic nerve

After training with a neuroradiologist with more than 8 years of experience (M.S.), OC and optic nerve measurements were performed by V.J. (radiology trainee), blinded to clinical data, using a standardized protocol: First, the central point of the OC was determined on all 3 planes. The axes of the planes were reoriented to the course of the optic pathway, so that they were perpendicular to the orientation of the individual OC in the central point. On the individually reoriented transversal plane, OC area, heights, and width were measured. Optic nerve diameters were measured in the cisternal segment 7 mm anterior to the previously defined central point of the OC perpendicular to the optic nerve course (Figs. 1 and 2). All measurements were performed using region-of-interest software from Horos, version 3.3.2 (https://www.horosproject.org). Width was defined as the diameter along the adjusted frontal axis. Heights were measured perpendicularly to that diameter: central height at the middle, the lateral heights at the maximal diameter left and right to the center. For interrater reliability analysis, J.V. and M.C. (trainee) measured OC dimensions within 10 randomly selected HC and intraclass correlations (ICC) were calculated.

### Clinical assessment

Neurological disability was on the Expanded Disability Status Scale (EDSS), including the visual functional system score according to the Neurostatus definitions [34]. Raters were under the supervision of board-certified neurologists. The global neurological examination also included assessment of ON history using clinical criteria. Visual acuity was tested monocularly under phototopic conditions using retroilluminated Early Treatment in Diabetes Retinopathy Study charts at a 4-m distance. The logarithm of the minimum angle of resolution (logMAR) served as a measure of visual function. Visual acuity data was included only from patients where best correction was used (*n* = 30).

### Optical coherence tomography measures

All OCT data were acquired on a spectral domain OCT device (Spectralis, Heidelberg Engineering) with automated real-time function. No pupil dilatation was used. We report the OCT acquisition settings and scanning protocol according to the APOSTEL recommendations [35]: The pRNFL thickness was measured using 3.4-mm ring scans around the optic nerve head (12°, 1536 A-scans, 9 ≤ ART ≤ 100). The GCIPL volume was measured using a 6-mm diameter cylinder around the fovea from a macular volume scan (25°×30°, 61 vertical B-scans, 768 A-scans per B-scan, ART = 15). Segmentation of the pRNFL and the intraretinal layers in the macular scan was performed semi-automatically using software provided by the optical coherence tomography manufacturer (Eye Explorer 1.9.10.0 with viewing module 6.0.9.0; Heidelberg Engineering). Quality was evaluated according to the OSCAR-IB criteria [36, 37].

Two patients did not have OCT data. Eight eyes from six NMOSD-ON had to be excluded due to incidental findings or quality reasons. Only the macular scan from two additional NMOSD-ON eyes was excluded due to quality reasons.

### Statistics

Proportional group differences were tested with *χ*^2^ test for sex and with ANOVA test for age. For comparison of ordinal and continuous measurements, groupwise comparison was performed using Kruskal-Wallis and ANOVA tests, respectively. Group comparison of OC dimensions was corrected for multiple comparison using the Holm-Bonferroni method. The variations of the individual metrics were compared within the HC group using a coefficient of variation (CoV) computed according to

$$ \mathrm{CoV}=\kern0.5em \frac{\mathrm{standard}\ \mathrm{deviation}\ \mathrm{of}\ \mathrm{the}\ \mathrm{individual}\ \mathrm{metric}}{\mathrm{mean}\ \mathrm{of}\ \mathrm{the}\ \mathrm{individual}\ \mathrm{metric}} $$

and served as an indicator of variation in the individual OC measures. Sensitivity to optic nerve atrophy of individual OC measures was evaluated with receiver operating characteristic (ROC) analysis, including area under the curve (AUC) comparison using DeLong method [38]. Association analysis of individual OC measures with the T2 lesion load, the SIENAX V-scaling [39] factor for head size and gender as potential influencing factors, mean optic nerve diameter, mean pRNFL thickness, mean GCIPL volume, visual function (mean logMAR), and disease duration was performed with the Pearson correlation test, association with the number of ON attacks with the Spearman test.

Statistical analyses were performed using R software, version 3.5.1. (http://www.r-project.org/) with the tidyverse [40], ggpubr [41], and pROC packages [42]. Statistical significance was set at a *p* value < 0.05.

## Results

### Demographics

Table 1 shows the demographic and clinical characteristics of the cohort. No significant differences of sex distribution, age, and physical disability were found between groups. Optic nerve diameters were different in NMOSD-ON compared to HC (*p* < 0.0001), NMOSD-ON compared to NMOSD-NON (*p* < 0.01), but not in NMOSD-NON compared to HC (*p* > 0.05).

### Group comparison and receiver operating characteristics

**Fig. 3:**
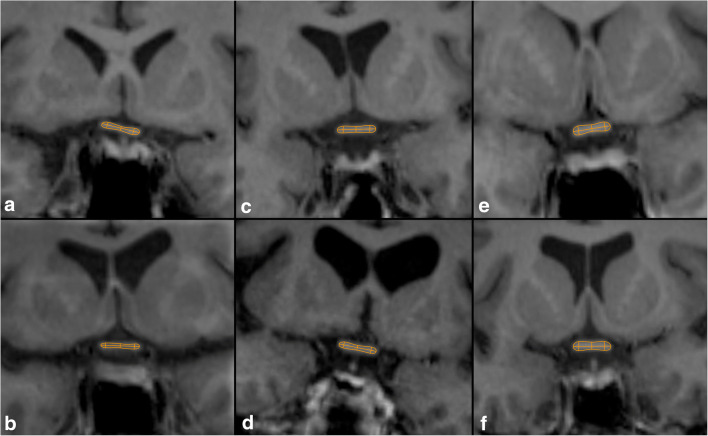
Difference in optic chiasm dimensions. Shown are OCs of NMOSD-ON (**a**, **b**), NMOSD-NON (**c**, **d**), and HC (**e**, **f**)

Figure 3 shows the OC of NMOSD-ON, NMOSD-NON, and HC to illustrate the reduction of OC dimensions in NMOSD.

**Fig. 4:**
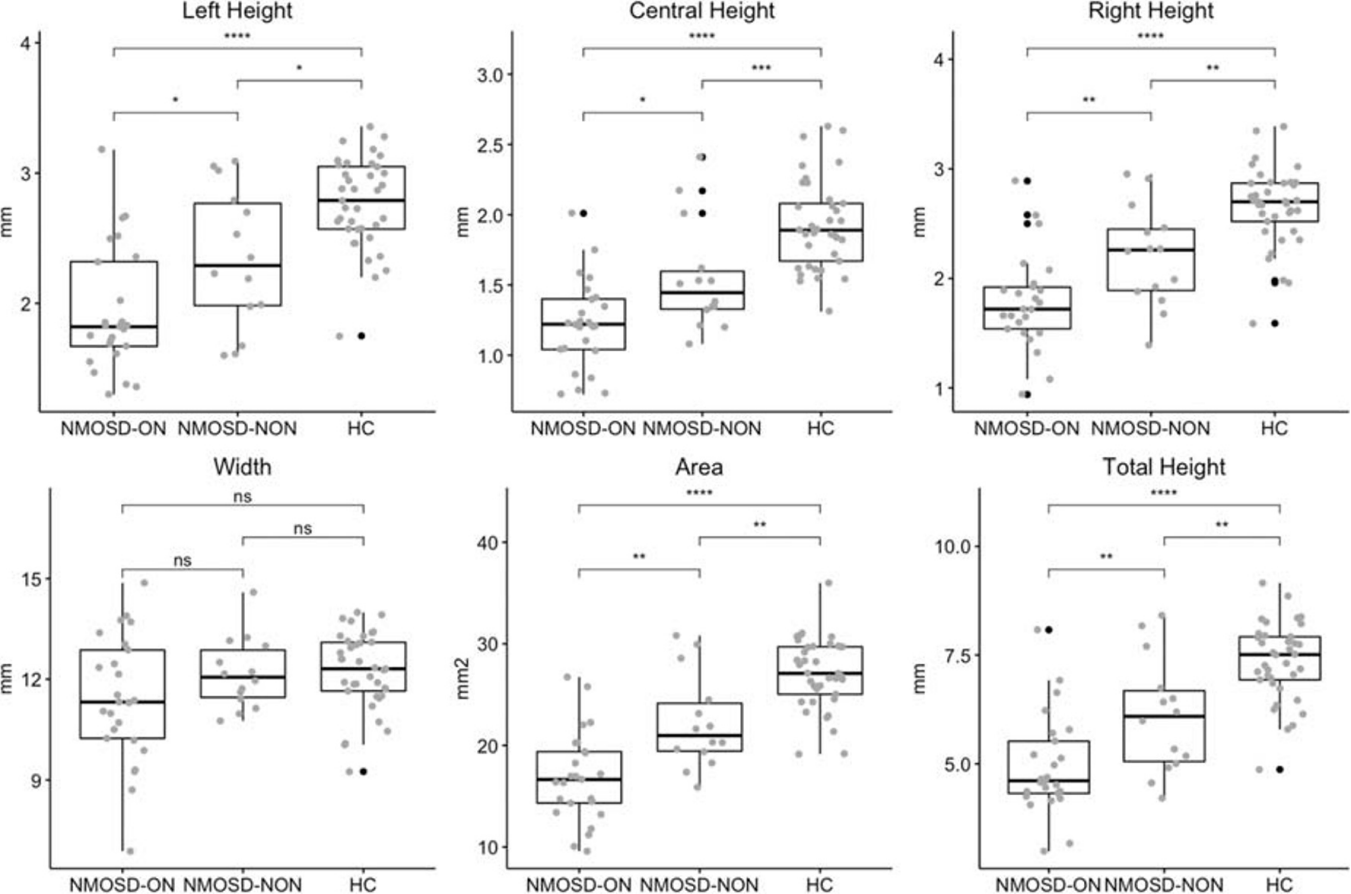
Optic chiasm measures. Group differences in optic chiasm heights, width, and area between neuromyelitis optica spectrum disorders patients with optic neuritis (NMOSD-ON), without optic neuritis (NMOSD-NON), and healthy controls (HC). ns: *p* > 0.05, * *p* ≤ 0.05, ** *p* ≤ 0.01, *** *p* ≤ 0.001, ****p ≤ 0.0001

All OC measures except width were significantly smaller in all group comparisons (NMOSD-ON vs. HC: *p* < 0.0001; NMOSD-NON vs. HC: *p* < 0.01; and NMO-ON vs. NMO-NON: *p* < 0.03), as shown in Table 2 and Fig. 4. When correcting for multiple comparisons (corrected *p* = 0.003), this remained significant for all measures for HC vs. NMOSD-ON, for all measures except left height for HC vs. NMOSD-NON and for area for NMOSD-NON vs. NMOSD-ON.

**Table 2:** Optic chiasm measures

Measure	HC	NMOSD-NON	NMOSD-ON	HC vs. NMOSD-NON	HC vs. NMOSD-ON	NMOSD-NON vs. NMOSD-ON	CoV	ICC
Left height (CI, mm)	2.77 (0.35)	2.34 (0.53)	1.94 (0.48)	*t* = 2.77 *p =* 0.01	*t* = 7.49 *p <* 0.0001	*t* = 2.36 *p =* 0.03	0.12	0.71
Central height (CI, mm)	1.93 (0.32)	1.55 (0.39)	1.22 (0.32)	*t* = 3.27 *p <* 0.001	*t* = 8.63 *p* < 0.0001	*t* = 2.70 *p =* 0.013	0.16	0.51
Right height (CI, mm)	2.65 (0.36)	2.20 (0.46)	1.79 (0.43)	*t* = 3.31 *p =* 0.003	*t* = 8.23 *p <* 0.0001	*t* = 2.79 *p =* 0.008	0.14	0.77
Width (CI, mm)	12.23 (1.15)	12.17 (1.05)	11.43 (1.87)	*t* = 0.17 *p =* 0.56	*t* = 1.91 *p =* 0.059	*t* = 1.59 *p =* 0.18	0.09	0.95
Area (CI, mm^2^)	27.07 (3.50)	22.26 (4.65)	16.89 (4.44)	*t* = 3.51 *p =* 0.003	*t* = 9.61 *p <* 0.0001	*t* = 3.51 *p =* 0.001	0.13	0.89
Total height (CI, mm)	7.35 (0.90)	6.09 (1.33)	4.94 (1.14)	*t* = 3.27 *p =* 0.003	*t* = 8.86 *p <* 0.0001	*t* = 2.73 *p =* 0.009	0.12	0.76

Shown are means of OC measures. *p* and *t*-values are derived from *t* tests. Corrected *p* = 0.003. *CI* = 95% confidence interval; *HC* = healthy controls; *NMOSD-ON* = neuromyelitis optica patients with history of optic neuritis; *NMOSD-NON* = neuromyelitis optica patients without history of optic neuritis; *CoV* = coefficient of variation; *ICC* = intraclass correlations

OC measures were not associated with the T2 lesion load (*r* = − 0.35, *p* > 0.09), the number of ON attacks (*r* = − 0.31, *p* > 0.13), the number of ON attacks per side (*r* = − 0.09, *p* > 0.13), the SIENAX V-scaling factor, or gender (*r* < 0.08, *p* > 0.23). Thus, no correction for head size or sex was performed.

**Fig. 5:**
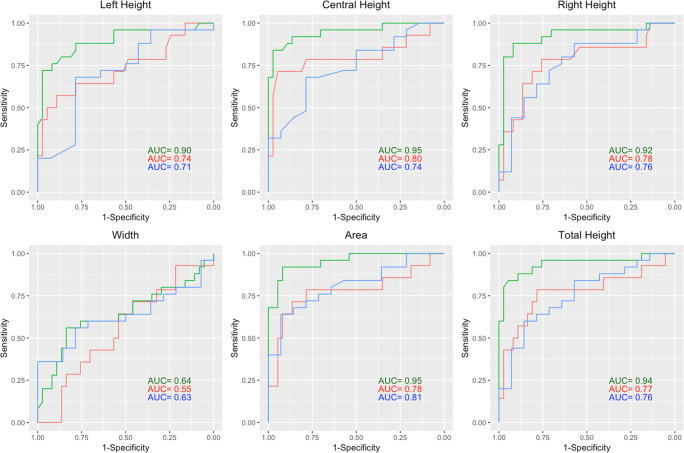
Receiver operating characteristic for optic chiasm measures. Each line represents one group differentiation: green lines HC vs. NMOSD-ON, red lines HC vs. NMOSD-NON, blue lines NMOSD-ON vs. NMOSD-NON; ON = optic neuritis

A ROC analysis was conducted to test the ability of the OC to predict the presence of damage in the anterior optic pathway, namely to differentiate between groups. OC area and OC heights have comparable AUC values for each group (NMOSD-ON vs. HC: AUC > 0.92; NMOSD-NON vs. HC: AUC > 0.74; NMO-ON vs. NMO-NON: AUC > 0.71), whereas width has lower AUC values, as shown in Table 3 and Fig. 5. AUC comparison using DeLong method and variation comparison using the CoV of the best performing measures revealed no significant difference. An OC area smaller than 22.5 mm^2^ yielded a sensitivity of 0.92 and a specificity of 0.92 in separating chiasms of NMOSD-ON from HC.

**Table 3:** Receiver operating characteristics analysis

**HC vs. NMOSD-ON**	**Area**	**Width**	**Left** **Height**	**Central** **Height**	**Right** **Height**	**Total** **Height**
AUC	0.95	0.64	0.90	0.95	0.92	0.94
95% CI	0.91–1.00	0.49–0.79	0.81–0.98	0.89–1.00	0.85–1.00	0.87–1.00
*p* value AUC comparison	–	< 0.01	0.02	0.78	0.16	0.40
**HC vs. NMOSD-NON**	**Area**	**Width**	**Left** **Height**	**Central** **Height**	**Right** **Height**	**Total** **Height**
AUC	0.78	0.55	0.74	0.80	0.78	0.77
95% CI	0.57–0.92	0.38–0.73	0.52–0.88	0.59–0.95	0.62–0.94	0.60–0.94
*p* value AUC comparison	–	0.08	0.31	0.49	0.93	0.77
**NMOSD-ON vs. NMOSD-NON**	**Area**	**Width**	**Left** **Height**	**Central** **Height**	**Right** **Height**	**Total** **Height**
AUC	0.81	0.63	0.71	0.74	0.76	0.76
95% CI	0.69–0.95	0.46–0.80	0.54–0.89	0.59–0.90	0.57–0.90	0.60–0.92
*p* value AUC comparison	–	0.08	0.06	0.19	0.29	0.14

Shown are area under the curve (AUC), 95% confidence interval (CI), and *p* value for AUC comparison using area as reference. *HC* = healthy controls; *NMOSD-ON* = neuromyelitis optica patients with history of optic neuritis; *NMOSD-NON* = neuromyelitis optica patients without history of optic neuritis

### Associations with structural and clinical measures

Table 4 summarizes the association analysis within the NMOSD-ON group. Higher OC measures were associated with bigger optic nerve diameter, better visual acuity, and better OCT measures. This was most prominent for OC area: Higher values significantly correlated with bigger optic nerve diameter (*r =* 0.4, *p* = 0.047), better logMAR (*r* = − 0.57, *p =* 0.013), thicker pRNFL (*r* = 0.59, *p = 0*.003), bigger GCIPL (*r* = 0.55, *p* = 0.007), and shorter disease duration (*r* = − 0.5, *p* = 0.012). Within OC heights, only central height was significantly associated with GCIPL (*r* = 0.46, *p =* 0.028).

**Table 4:** Associations of OC measures and visual acuity, optic nerve diameter and optical coherence tomography measures for NMOSD-ON patients

Mean	Area	Width	Left Height	Central Height	Right Height	Total Height
Visual acuity (logMAR)	*r* = − 0.57 *p =* 0.013	*r* = − 0.53 *p =* 0.023	*r* = − 0.05 *p =* 0.84	*r* = − 0.43 *p =* 0.079	*r* = − 0.21 *p =* 0.41	*r* = − 0.22 *p =* 0.39
Optic nerve diameter	*r* = 0.4 *p =* 0.047	*r* = 0.75 *p <* 0.001	*r* = − 0.06 *p =* 0.78	*r* = − 0.09 *p =* 0.68	*r* = 0.09 *p =* 0.66	*r* = − 0.03 *p =* 0.87
pRNFL thickness	*r* = 0.59 *p =* 0.003	*r* = 0.54 *p =* 0.008	*r* = 0.15 *p =* 0.49	*r* = 0.38 *p =* 0.07	*r* = 0.30 *p =* 0.16	*r* = 0.28 *p =* 0.19
GCIPL volume	*r* = 0.55 *p =* 0.007	*r* = 0.33 *p =* 0.13	*r* = 0.19 *p =* 0.39	*r* = 0.46 *p =* 0.028	*r* = 0.34 *p =* 0.11	*r* = 0.33 *p =* 0.12

*r* values are Pearson’s correlation coefficients. *NMOSD-ON* = neuromyelitis optica patients with history of optic neuritis; *logMAR* = logarithm of the minimum angle of resolution; *pRNFL* = peripapillary retinal nerve fiber layer; *GCIPL* = combined ganglion cell-inner plexiform layer

## Discussion

We evaluated OC measures as imaging marker of anterior optic pathway damage. We demonstrated significant group differences between NMOSD patients and HC and strong associations of OC measures with structural and clinical measures. Our data show that OC assessment in standard 3D-T1w images is sensitive to anterior optic pathway damage. Hence, OC measures are easily accessible and sensitive markers of anterior optic pathway damage.

OC dimension values for HC presented in our study are similar to recently published data [33]. A previous study by Wagner et al [31], however, reported slightly higher OC width and area values. This study excluded OC height due to a high degree of variance. Notably, unlike our study, Wagner et al did not account for the transverse course of the optic pathway and did not define precise measure locations. Thus, the coronal plane would be at varying angles (not perpendicular) to the course of the optic pathway at which OC assessment results in higher values and variance. Furthermore, our study employed a higher resolution MRI sequence, which may have an impact on values and variance.

OC heights and area showed differences between all groups. This is in line with earlier investigations suggesting that optic nerve dimensions discriminate ON patients from controls [16]. OC assessment, as suggested in our study, only requires a standard and broadly available MRI sequence (3D T1-weighted MPRAGE) and assesses a rather fixed structure less vulnerable to motion artifacts compared to the previously used orbital optic nerves [3, 4, 16, 17]. Note that in this study optic nerve diameters were measured in the cisternal segment 7 mm anterior to the OC, since contrast heterogeneity and motion artifacts in the orbital part rendered orbital assessment difficult in 25% of the patients. The observation that smaller OC dimensions were also found in NMOSD-NON (compared to HC and NMOSD-ON) supports microstructural changes in the optic pathway independent of ON [26], which have been described in NMOSD [5, 27, 43, 44]. In concordance with a study by Harrigan et al [16], the optic nerve diameter was smaller in patients with a history of ON compared to HC but not in patients without ON. Although this should not be overstated in consideration of the small NMOSD-NON sample size, this might indicate that microstructural changes independent of ON, including anterograde degeneration originating from anterior optic pathway damage but also retrograde degeneration originating from posterior optic pathway damage, might accumulate in the OC.

The AUC values obtained from ROC analyses for OC area and heights indicate that OC measures are sensitive to anterior optic pathway damage. On the descriptive level, OC left height shows slightly poorer ROC performance than right. This might rather be caused by an asymmetric severity of atrophy predominantly affecting fibers contributing to the right side of the OC (8 ON right vs. 6 ON left), of handedness or other asymmetries present in our study, than by a different sensitivity of the individual measure. This and the observation that no association between ON attacks per side and OC heights was found highlight the fact that OC assessment accumulates pathophysiologic processes of both sides and does not provide information on the origin of the fibers.

The observed associations between higher OC area and better visual acuity (*r* = − 0.57), thicker pRNFL (*r* = 0.53) and bigger GCIPL (*r* = 0.55) in NMOSD are similar to the associations reported on optic nerve dimensions (*r* = − 0.50, *r* = 0.66, *r* = 0.59) in MS [17]. The degree of association between pRNFL and anterior optic pathway dimensions in MRI might be equally limited in NMOSD and MS, since axonal loss is not the only substrate of neuronal atrophy and myelin loss, gliosis, and changes in water content also contribute to MRI-detected changes after ON [45]. Alongside findings suggesting that axonal loss is a major substrate of MRI-detected optic pathway atrophy after ON [17–20], the association between pRNFL (a surrogate for retinal axons) and OC measures implies that they are sensitive to atrophic changes of the anterior optic pathway. The association of OC area with visual function suggests a role for the OC as an imaging marker of neurodegenerative damage in the optic pathway with potential functional relevance.

Despite MRI’s broad availability, no standardized MRI method for evaluation of optic pathway degeneration in standard scans is available. One major problem in optic nerve assessment is defining standardized measurement locations along the variable course of the nerve, which has high inter-individual variability even in healthy populations [3, 4, 15, 17]. Several methods to measure optic nerve dimensions have been put forward [16, 17, 32]. These methods typically involve dedicated orbital MRI fat-saturated acquisition sequences along the axis of the optic nerve [3] additional to the commonly acquired sequences and extend the scan time for each patient. Others involve complex imaging post-processing procedures [16] and, thus, may be difficult to implement in the routine clinical workup. Moreover, motion artifacts from eye movements and contrast reduction in the posterior region of the optic nerve, due to thinning of the CSF filled sub-arachnoid space, render MRI-based optic nerve assessment technically difficult [16]. This limits the accessibility of optic nerve measurements using MRI in the clinical setting.

The OC is less vulnerable to motion artifacts and consistently surrounded by CSF. It is less variable in morphology, bigger in dimensions and, thus, a simple target for MR investigations. OC assessment, as suggested in our study, only requires a broadly available MRI sequence (3D T1-weighted). While it does not provide information on the origin of the fibers and evaluation of focal optic nerve damage might better be achieved by direct measurement at the sight of inflammation, it accumulates neurodegeneration from both sides of the anterior optic pathway causing observable impairment. Thus, it extends the amount of information from a single measurement in the conventional and clinical standard scan, which can be used for monitoring of disease progression or therapeutic effectiveness.

Despite the low total number of subjects included in the study owing to the low prevalence of the disease, a significant difference in OC measures was shown within a relatively large homogeneous cohort exclusively consisting of AQP4-IgG-positive NMOSD patients. Separate gender analysis could not be conducted due to the high proportion of female patients. This is in line with the strong female preponderance in NMOSD [46]. OC width was measured along straight lines, which may not account for curved width. In future investigations, curved lines could be drawn; however, only few participants showed recognizable deviation from straight lines and, thus, we do not expect that this would drastically change the presented results.

Our data provide a strong rationale for future, larger studies on OC measures in ON, including in NMOSD patients with acute ON, in which inflammation might result in an increase in OC dimensions, as well as in patients with inflammatory diseases such as MS and myelin oligodendrocyte glycoprotein antibody associated disease (MOGAD) [47]. Finally, studies on the influence of susceptibility artifacts on scanners with different field strength and resolution seem justified and the application of advanced quantitative imaging methods such as DTI could reveal insights into the relationship between anterior and posterior optic pathway neurodegeneration.

## Conclusion

Our study represents an initial and thorough assessment of OC measures to evaluate optic pathway degeneration using standard MRI and shows that the OC area is suitable and reliable. This simple method extends the amount of information that can be obtained from conventional and clinically available scans. Our results suggest that the OC might evolve into an easily accessible imaging marker of neurodegeneration in the anterior optic pathway with potential functional relevance.

## Abbreviations

AQP4-IgG: Aquaporin 4-IgG
AUC: Area under the curve
CI: 95% confidence interval
CoV: Coefficient of variation
EDSS: Expanded Disability Status Scale
GCIPL: Combined ganglion cell-inner plexiform layer
HC: Healthy controls
ICC: Intraclass correlations
logMAR: Logarithm of the minimum angle of resolution
NMOSD: Neuromyelitis optica spectrum disorders
NMOSD-ON: Neuromyelitis optica patients with history of optic neuritis
NMOSD-NON: Neuromyelitis optica patients without history of optic neuritis
OC: Optic chiasm
OCT: Optical coherence tomography
ON: Optic neuritis
pRNFL: Peripapillary retinal nerve fiber layer
ROC: Receiver operating characteristics
